# Pathology and Molecular Biology of Melanoma

**DOI:** 10.3390/cimb45070352

**Published:** 2023-06-30

**Authors:** Tanase Timis, Jon Thor Bergthorsson, Victor Greiff, Mihai Cenariu, Diana Cenariu

**Affiliations:** 1 Department of Oncology, Bistrita Emergency Hospital, 420094 Bistrita, Romania; timis_tanase@yahoo.com; 2 Department of Hematology, Iuliu Hatieganu University of Medicine and Pharmacy, 400347 Cluj-Napoca, Romania; 3 Department of Pharmacology and Toxicology, Medical Faculty, University of Iceland, Hofsvallagotu 53, 107 Reykjavík, Iceland; jon.bergthorsson@gmail.com; 4 Department of Immunology, University of Oslo, Oslo University Hospital, 0372 Oslo, Norway; victor.greiff@medisin.uio.no; 5 Department of Animal Reproduction, University of Agricultural Sciences and Veterinary Medicine, 3-5 Calea Manastur Street, 400372 Cluj-Napoca, Romania; mihai.cenariu@usamvcluj.ro; 6 Medfuture Research Center for Advanced Medicine, Iuliu Hatieganu University of Medicine and Pharmacy, 400337 Cluj-Napoca, Romania

**Keywords:** melanoma, molecular biology, genetics, clinical implications

## Abstract

Almost every death in young patients with an advanced skin tumor is caused by melanoma. Today, with the help of modern treatments, these patients survive longer or can even achieve a cure. Advanced stage melanoma is frequently related with poor prognosis and physicians still find this disease difficult to manage due to the absence of a lasting response to initial treatment regimens and the lack of randomized clinical trials in post immunotherapy/targeted molecular therapy settings. New therapeutic targets are emerging from preclinical data on the genetic profile of melanocytes and from the identification of molecular factors involved in the pathogenesis of malignant transformation. In the current paper, we present the diagnostic challenges, molecular biology and genetics of malignant melanoma, as well as the current therapeutic options for patients with this diagnosis.

## 1. Background on Melanoma

Melanoma is a type of cancer that originates from the uncontrolled growth of melanocytes. While it can occur in various parts of the body, including the mucosal surfaces, uveal tract, and leptomeninges, this review will concentrate on cutaneous melanoma. Cutaneous melanoma is the leading cause of death among skin cancers and its incidence is continuously increasing. The World Health Organization (WHO) reports that it is the fifth most common cancer in men and the sixth most common in women, with an estimated 325,000 new cases worldwide in 2020 [1].

Although it accounts for only 10% of skin cancers, it causes over 90% of skin cancer deaths. It affects people of all ages, with a median age at diagnosis of 63 years, and is the leading cause of cancer deaths in people aged 25–29 [2].

The incidence of melanoma is rising in younger women. One study found a 50% increase in melanoma cases among women aged 18–39 between 1970 and 2009, while another found that melanoma incidence was increasing faster in young women than in young men, with the largest increase seen in women aged 25–29 [3,4]. Several factors may contribute to the rising incidence of melanoma in young women, such as ozone depletion, high levels of pollution and climate change, increasing use of tanning beds, and lack of awareness about skin cancer prevention and the importance of using protective measures such as sunscreen and protective clothing [5].

Avoiding exposure to the sun at peak hours and using protective clothing, sunglasses, and sunscreen with a minimum SPF of 15 are the main ways to prevent melanoma. The most important prevention measure is preventing sunburn during childhood and adolescence. Other risk factors include atypical moles, a large number of benign moles, a family history of melanoma, and a skin type that is prone to burning. Germline mutations in the CDKN2A gene are the most common cause of hereditary melanoma, accounting for about 20–40% of melanoma cases in families. However, the majority of melanomas (90%) are sporadic. Skin melanomas have the highest average number of somatic mutations of all tumors. The most common are activating mutations in the BRAF gene (especially BRAF V600E, followed by BRAF V600K), which are found in 50% of patients and are more common in superficial spreading melanoma. Mutations in the NRAS gene are found in 15–20% of melanomas, while mutations in the NF1 gene (neurofibromin-1) are found in 15% of melanomas. Mutations in the KIT gene (found in mucosal, acral, and lentigo-malignant melanomas) are present in about 10% of patients. Uveal melanomas are more commonly associated with mutations in the GNAQ/GNA1 genes [6].

Though melanoma incidences have seen a recent increase, the disease has a long history and has been documented in the past. The first recorded case of melanoma dates back to the 17th century, where doctors described fatal black tumors spreading in patients. In the following years, the only treatment for primary melanoma was surgical removal and lymph node excision. It was only in the 1940s, with the advent of chemotherapy and its application in treating cancer, that there was a small improvement in the treatment of melanoma [7].

One of the first chemotherapy drugs for melanoma was dacarbazine, introduced in the 1970s. A 1976 case series published in Cancer reported its effectiveness, with some patients showing partial or complete responses. Since then, dacarbazine has mostly been used in combination with other chemo drugs or targeted therapies like BRAF inhibitors. Its effectiveness as a standalone treatment is limited.

In 1995, interferon alpha became the first immunotherapy approved for melanoma treatment in the US, following the Phase III ECOG 1684 trial. The trial compared Interferon alpha to a placebo and showed its effectiveness in treating melanoma. In 2011, Vemurafenib, a BRAF inhibitor, was approved by the FDA based on the BRIM-3 study, which showed it was more effective than dacarbazine chemotherapy. In 2013, dabrafenib, another BRAF inhibitor, and trametinib, an MEK inhibitor, were approved for melanoma treatment based on the BREAK-3 and METRIC trials, respectively. The same year, Ipilimumab, the first immune checkpoint inhibitor, was approved for advanced melanoma based on the MDX010-20 study. Pembrolizumab and nivolumab have been authorized for advanced melanoma since 2014 based on the KEYNOTE-001 and CheckMate-066 studies, respectively, and now are used as standard treatments for advanced melanoma and other cancers [8,9].

The field of melanoma research thus continues to advance with promising new therapies under development. Under current investigation, promising cancer therapies include adoptive cell transfer, oncolytic viruses, CAR-T cells, MAGE-A3 cancer immunotherapeutic, immunogenic cell death, and MicroRNAs as potential therapeutic targets.

Oncolytic viruses are viruses that are specifically designed to infect and kill cancer cells while sparing normal cells. T-VEC (talimogene laherparepvec) is an example of oncolytic immunotherapy used in advanced melanoma. OPTiM (Oncovex Pivotal Trial in Melanoma) was a Phase III randomized clinical trial that evaluated the efficacy and safety of T-VEC compared to granulocyte-macrophage colony-stimulating factor (GM-CSF) in patients with unresectable stage IIIB-IV melanoma. Based on the results of this trial, the FDA approved talimogene laherparepvec (T-VEC) in October 2015 for the treatment of unresectable stage IIIB-IV melanoma [10]. 

CAR-T cells are a type of immune cell that has been genetically modified to recognize and attack cancer cells. Researchers are investigating the use of CAR-T cells as a treatment for melanoma.

MAGE-A3 cancer immunotherapeutic is also of potential future use, as MAGE-A3 is a protein that is expressed by many types of cancer cells, but not by normal cells. Researchers are developing a vaccine that targets MAGE-A3 in order to stimulate the immune system to attack cancer cells. See Table 1.

Combination therapy represents an active area of research in melanoma treatment.

Using multiple treatment modalities simultaneously or sequentially improved outcomes compared to single-agent therapies.

Some combination therapies are already approved, but other molecule combinations are still under investigation, and ongoing research aims to optimize their efficacy and minimize potential side effects.

Immune checkpoint inhibitors, such as anti-PD-1 antibodies (e.g., pembrolizumab, nivolumab) and anti-CTLA-4 antibodies (e.g., ipilimumab) have shown improved response rates and survival outcomes compared to monotherapy. The combination of ipilimumab and nivolumab has been approved as a first-line treatment for unresectable or metastatic melanoma from 2015.

Combining targeted therapy with radiotherapy has shown synergistic effects in melanoma treatment. Targeted therapy can sensitize tumor cells to radiation, leading to enhanced tumor control and improved survival outcomes.

While traditional chemotherapy has limited effectiveness in melanoma, combining chemotherapy with immunotherapy or targeted therapy in certain cases may provide added benefit.

The rationale behind this combination is that chemotherapy can help create a more favorable tumor microenvironment and enhance the immune response to immunotherapy. Ipilimumab with temozolomide in patients with metastatic melanoma demonstrated promising clinical activity, with an objective response rate of 16.7% and durable responses observed in some patients [26].

New combination therapies are using anti-PD-1/PD-L1 antibodies with anti-LAG-3 (lymphocyte-activation gene 3) antibodies.

Combining the anti-LAG-3 antibody with nivolumab in patients with advanced solid tumors, including melanoma, showed promising antitumor activity and warranted further investigation in larger clinical trials [20].

Another future direction in melanoma treatment is the combination of miRNAs with immunotherapy or targeted therapy. Preclinical studies investigated the combination of miR-34a with ipilimumab in melanoma. The findings demonstrated that the combination of miR-34a and ipilimumab synergistically enhanced antitumor immune responses and improved survival outcomes in mouse models of melanoma. MiR-34a could potentially be used as an adjuvant therapy to enhance the efficacy of immune checkpoint inhibitors in melanoma treatment [27].

Another study focused on the combination of miR-34a with vemurafenib.

The results showed that the combination therapy synergistically inhibited melanoma cell proliferation and tumor growth in mouse models compared to single-agent treatment. The researchers suggested that combining miR-34a with targeted therapy could be a promising strategy to enhance the efficacy of targeted therapies in BRAF-mutant melanoma [22].

The field of melanoma treatment research is dynamic, and new discoveries and advancements continue to shape the future of treatment. Ongoing clinical trials and translational research are crucial for furthering our understanding and improving outcomes in advanced melanoma.

## 2. The Biology of Melanoma Dissemination

The spread, survival, and multiplication of cancerous cells from the initial tumor in distant anatomical areas is referred to as the metastatic process. According to the American Cancer Society, about 20% of people with melanoma have tumors that are metastatic at the time of diagnosis [3]. The route of a melanoma metastasis can be lymphatic, hematologic, or directly through the surrounding tissue.

The first person to describe melanoma metastasis was the German dermatologist Wilhelm Buschke in 1892. In his paper, Buschke described the case of a patient with melanoma of the eye (ocular melanoma) who had developed metastases in the liver. This was one of the first reports of melanoma metastasis in the medical literature. Since then, there have been countless studies of this process, but until this day, we inherit a linked knowledge gap regarding metastasis formation [7].

Successful metastasis is accomplished by the five key steps of the metastatic cascade: invasion, intravasation, circulation, extravasation, and colonization at secondary tumor sites. Malignant cells from the original tumor penetrate the basement membrane and surrounding stromal tissue before entering a blood or lymphatic vessel and being transported out by the circulation. They become circulating tumor cells (CTCs) in the bloodstream, and those that survive and reach distant organs become disseminated tumor cells (DTCs), which have the potential to form secondary tumors [28,29]. Cells enter the surrounding tissue as a group, a process known as “collective invasion”, or as individual cells, a process known as “single-cell invasion” during the local invasion phase.

In the latter scenario, cells may go through an epithelial to mesenchymal transition (EMT) in order to rupture the basement membrane (BM) [29].

Although a generally accepted term, EMT describes the above-mentioned processes in carcinomas, but expanding data suggests explicit roles of EMT-TFs also in non-epithelial malignancies, such as melanoma identified as phenotype switching. This is a more general but possibly more appropriate term when considering the EMT-like processes in melanoma.

Metastasis is typically seen as a mechanism driven by pleiotropic translation variables (TWIST, Snail, ZEB 1 and 2) which back epithelial cells to enter a mesenchymal state. After dissolving the basal film, tumor cells reach the stroma, where their harmful forcefulness is affected by an assortment of tumor-related stroma cells that are characteristic for each state of the tumor movement. Tumor cells that attack the stroma meet with fibroblasts, endothelial cells, adiposities, macrophages, and other cells of the immune structure. The intravasation occurs within the microvessels, by crossing the pericyte and endothelial cell wall. Neoplasm triggers neoangiogenesis and shapes capillaries in which endothelial cell connections are fragile and have a low pericyte coating of the walls. This process makes it more accessible for tumor cells to enter the circulation. Tumor neoangiogenesis and weak junctions between endothelial cells are directed by vascular endothelial growth factors (VEGF), cyclooxygenase-2 (COX-2), MMP1 and 2 [20,22,26,27,28,29,30]. Intravasation of tumor cells is decided by the transforming growth factor-β (TGF-β) that encourages malignant cells penetration of the microvessels barrier. Once cancer cells reach circulation, they ought to endure the hemodynamic pressure and the host’s immune system. They do so by shaping tumor cell clusters and co-opting platelets, essentially coating themselves for safety. The location where a CTC can halt is more often set by the arrangement of the blood circulation within the body. Once entangled, CTCs either enter a latent state that can last from months to decades, or begin colonizing the new area instantly. Notably, the dormancy state is connected with cell plasticity in NRAS- and BRAF-mutated melanomas that lead to increased survival or therapy resistance. From the microvessel in which they ended up captured, tumor cells can extravasate by developing intraluminal, breaching the barrier of the microvessels, and getting in intimate contact with organ parenchyma, or individual cells can pass through the gaps of the endothelium and pericytes [30,31,32,33,34,35,36].

The environment where tumor cells end up entangled varies from that of the primary location. A hypothesis through which DTCs adjust to the actual environment hypothesizes that tumor cells produce a premetastatic niche by altering the environment to better fit their needs.

This new environment requires new oncometabolism, which is also a precious area of research. There is evidence that metastatic microenvironment oncometabolism is regulated by miRNAs. For example, miRNAs such as miR-25, miR-125b, and miR-155 have been found to be upregulated in melanoma, and they also regulate the expression of enzymes involved in metabolic pathways. In addition, miRNAs have been found to adjust the expression of transcription factors that are involved in the regulation of oncometabolism, such as HIF1α and c-Myc. These transcription factors are known to be important regulators of cancer cell metabolism and are often dysregulated in melanoma. Overall, the role of miRNAs in the regulation of oncometabolism in melanoma is an active area of research, and further studies are needed to fully understand the mechanisms underlying this process and to identify potential therapeutic targets.

Spread tumor cells can shape into micrometastases that can finally form viable metastases. The larger part of tumor cells either regresses or remains latent within the host tissue, which besides the annihilation in the blood stream, makes the mechanism of metastasis ineffective. Additionally, single disseminating cells are less successful than clusters (implying some sort of cohesion). Cohesion between melanoma cells is mediated by N-cadherin, αvβ3-integrin, L1-CAM, AL-CAM, and MCAM/MUC18, that are not expressed on melanocytes. This is also true for melanoma, which is known for its aggressive behavior and high propensity for metastasis. However, despite its aggressive nature, melanoma remains an inefficient metastatic colonizer [30,31,32,33,34,35,36].

MMP-2 (matrix metalloproteinase-2) and MMP-9 (matrix metalloproteinase-9) have also been studied in the context of melanoma progression and metastasis. These two MMPs are among the most well-studied members of the matrix metalloproteinase family in relation to melanoma. MMP-2 and MMP-9 are metalloproteinases, which means they are capable of degrading type IV and type V collagens, key components of the extracellular matrix. They are involved in the breakdown of the extracellular matrix, promoting tumor cell invasion, angiogenesis (the formation of new blood vessels), and metastasis.

Several studies have shown that the expression and activity of MMP-2 and MMP-9 are increased in melanoma cells compared to normal skin cells. This upregulation has been associated with a more invasive and metastatic phenotype in melanoma. 

MMP-2 and MMP-9 are secreted by both melanoma cells and stromal cells in the tumor microenvironment. They degrade the extracellular matrix, allowing melanoma cells to invade surrounding tissues, penetrate blood vessels or lymphatic vessels, and disseminate to distant sites in the body. In addition to their role in promoting invasion and metastasis, MMP-2 and MMP-9 have also been implicated in other aspects of melanoma progression. They can influence angiogenesis by promoting the release of pro-angiogenic factors and the remodeling of blood vessels within the tumor. Moreover, they contribute to immune evasion by degrading components of the immune system, enabling melanoma cells to evade detection and destruction by the immune system [37].

There are many factors that can contribute to the efficiency of metastatic colonization, including the capacity of cancer cells to outlive within the systemic circulation, the ability to overcome immune surveillance, and the strength to adapt to and thrive in the microenvironment of the distant site.

## 3. Diagnostic Pathology of Melanoma

The histological features of melanomas imitate those of lymphomas, sarcomas, neuroendocrine tumors, and Merkel cell carcinomas; for instance, both express epithelial cytokeratin 20 and endothelial markers, thus making melanomas a serious challenge, even for an experienced pathologist.

Immunohistochemical workup for molecular markers is a vital step for the diagnosis of malignant melanoma, but moreover, in staging, evaluating prognosis, treatment management, and predicting recurrence. Current molecular data recommends that melanoma should be assessed as a heterogeneous group of lesions with distinct alteration at the molecular level that includes changes of cellular mechanisms like cell signaling, cell differentiation, cell adhesion, and apoptosis [8,38]. 

There are several proteins that are commonly used as markers for melanoma in immunohistochemistry (IHC) testing.

These include the S-100 protein, with an almost 100% sensitivity for melanoma, HMB-45 (also found in melanocytes, and also used in combination with the S-100 protein to confirm the diagnosis of melanoma), MART-1 (also found in melanocytes and is used in combination with the S-100 protein and HMB-45 to confirm the diagnosis of melanoma), BRAF protein (often found in melanoma cells and can be targeted with specific drugs, such as vemurafenib, which may be used to treat the melanoma), and KIT (also often found in melanoma cells and can be targeted with specific drugs, such as imatinib, which may be used to treat the melanoma).

Prognostic factors like ulceration and mitosis rate provide significant information regarding the aggressiveness of the tumor. Usually, ulceration and high mitosis rates are present in thick tumors, and indicate greater chances of finding positive lymph node metastases. This feature was evaluated by the frequency of mitoses detected for each category. A superior mitosis rate is connected to a fast tumoral size increase, impassive to the lesion’s dimensional characteristics. 

In cutaneous melanoma, the Breslow thickness is a critical characteristic, and the most important prognostic factor. Mitosis levels were shown to be substantially linked with Breslow depth.

S100 protein is a biomarker utilized in the assessment of tumors with a low degree of differentiation, with nearly 100% sensitivity for melanoma. The protein is involved in cellular division, calcium metabolism, protein phosphorylation and secretion, cellular growth, and the control of cellular proliferation. S100 has been shown to be expressed in a range of poorly differentiated cancers, as well as illnesses such as neurodegenerative disorders, inflammatory diseases, and cardiomyopathies. S100 has recently been linked to a variety of cancers, including melanoma [8,38].

There are different isoforms, or variations, of the S-100 protein that are produced by different cells in the body. For example, S-100A1, S-100A2, and S-100A4 are produced by melanocytes, and have been found to be elevated in some cases of melanoma. S-100B is produced by Schwann cells (a type of nerve cell) and has been found to be elevated in some cases of melanoma and other types of cancer. S-100P is produced by certain cells in the immune system and has been found to be elevated in some cases of melanoma and other types of cancer.

S100 subtypes: S100B, S100P, S100A4, S100A6, and S100A13 are often found in melanoma, with S100P being positive in all melanoma subtypes. The link is weaker in oral malignant melanoma than in cutaneous melanoma, with the former showing both a lower grade of S100 staining and a higher biological aggressiveness than the latter [8,38].

Ki67 is a cell cycle regulatory protein for which a particular antibody is used to confirm the presence of a nuclear antigen that is only found in tissues with a high rate of cellular proliferation, and is otherwise missing in normal tissue. The expression of Ki67 correlates with the progression of the disease: a greater Ki67 level is connected with thicker tumors and, as a result, a less positive prognosis for the patients.

HMB 45, which is the abbreviation for ‘human melanoma black’, was identified in 1986, and detects a melanosomal glycoprotein (Pmel17) involved in the formation of melanosomal fibrils and the transition from stage I to stage II premelanosomes. HMB 45 is a frequently utilized marker for the positive diagnosis of malignant melanoma and in the evaluation of sentinel lymph nodes to rule out the occurrence of micrometastases. In desmoplastic melanoma, HMB 45 staining is frequently negative.

Melan A, also known as MART 1, is a protein found in melanocytes that might be utilized as a histopathological marker to diagnose malignancies generated from melanocytic progenitors (Table 2).

It is a sensitive and specific marker for the diagnosis of melanoma, although it can also be present in other types of cancer with melanocytic lineage like clear cell sarcoma, benign nevi, melanotic neurofibroma, and perivascular epithelioid cell tumors [8,38].

Tyrosinase is also used as an immunohistochemical marker in the diagnosis of melanoma. Tyrosinase IHC staining can help confirm the diagnosis of melanoma and distinguish it from other types of skin cancers or benign lesions. 

Several studies evaluated the expression of tyrosinase in various types of melanoma and peripheral nerve tumors using immunohistochemistry. The researchers found that tyrosinase staining was strongly positive in 84% of melanoma cases, including both conventional and desmoplastic melanomas. In contrast, peripheral nerve tumors showed negative or weak staining for tyrosinase.

They also found that tyrosinase and Melan A were highly expressed in malignant melanoma cases, with tyrosinase showing strong staining in 89% of cases, and Melan A showing strong staining in 92% of cases. Spitzoid melanoma cases exhibited weaker staining for both markers [48].

## 4. Genetics of Melanoma

### 4.1. Germline Mutations

Germline mutations are genetic alterations that occur in the DNA of reproductive cells (eggs and sperm) and can be passed down from one generation to the next. These mutations are present in all cells of an individual’s body and can increase the risk of developing certain diseases, including melanoma.

CDKN2A, CDK4, and BRCA1-associated protein 1 (BAP1) have been identified as high-risk melanoma susceptibility genes. These genes are all carried in an autosomal dominant way, indicating vertical disease transmission and a similar number of afflicted men and women. Multiple high-risk susceptibility genes have been predicted to exist, but no additional genes have been confirmed to exist.

CDKN2A (also known as p16INK4a or p16) is a protein that plays a role in the cell cycle, the process by which cells grow and divide. CDKN2A acts as a tumor suppressor, meaning that it helps to regulate cell growth and prevent the development of cancer. Mutations or changes in the CDKN2A gene can lead to an impaired ability to suppress tumor growth, which can increase the risk of cancer. Studies have shown that CDKN2A mutations are more common in melanomas that have spread to other parts of the body (metastatic melanoma) than in localized melanomas that have not spread. CDKN2A mutations are more common in melanomas that develop in people with fair skin and in people who have a history of sun exposure.

In melanoma, CDKN2A mutations are often found in combination with other genetic changes, such as BRAF mutations. The presence of CDKN2A mutations can influence the prognosis and treatment of melanoma, and they are often taken into consideration when determining the appropriate course of treatment. Although CDKN2A mutations and its modulators increase the risk of melanoma, others like host, genetic, and environmental factors also increase that risk. The identification of these characteristics should aid in lowering the risk of melanoma in vulnerable individuals and families with mutations, as well as increasing the understanding of disease processes. Atypical/dysplastic nevi, higher numbers of typical nevi, and poor tanning ability and/or tendency to sunburn have all been found as host-modifying variables in melanoma-prone families with CDKN2A mutations. UV radiation has also been shown to modify melanoma risk in families with CDKN2A mutations [49].

One study identified a specific CDKN2A mutation known as p16-Leiden (c.225_243del19) in Dutch melanoma families. The p16-Leiden mutation was found to be highly prevalent in families with a history of melanoma and was associated with an increased risk of developing the disease.

Other studies found that patients with CDKN2A mutations had a poorer response to BRAF and MEK-targeted therapies or immune checkpoint inhibitors, such as anti-PD-1 antibodies, compared to those without these mutations [50].

The CDK4 gene encodes a protein called cyclin-dependent kinase 4, which plays a role in regulating the cell cycle. In normal cells, the cell cycle is carefully regulated, allowing cells to divide and grow in a controlled manner. However, in cancer cells, including melanoma cells, the cell cycle can become unregulated, leading to uncontrolled proliferation and the development of a tumor. In melanoma, CDK4 is often overexpressed, which can contribute to the uncontrolled proliferation of melanoma cells. 

CDK4 helps to drive the cell cycle forward by phosphorylating (adding a phosphate group to) certain proteins that are involved in cell cycle regulation. When CDK4 is overexpressed, it can lead to an excess of these phosphorylated proteins, which can result in an acceleration of the cell cycle and an increase in the proliferation of melanoma cells. CDK4 inhibitors have been studied as potential treatments for melanoma [51].

One phase 2 clinical trial highlighted the potential of CDK4 inhibitor ribociclib in combination with the MEK inhibitor binimetinib as a treatment approach in melanoma, particularly in cases with specific genetic alterations involving CDK4. The combination therapy showed promising efficacy, with a disease control rate of 70% and an objective response rate of 20% in the study population [52].

MC1R (melanocortin 1 receptor) is a protein that is found on the surface of melanocytes, the pigment-producing cells in the skin. MC1R plays a role in determining the type and amount of pigment produced by melanocytes, and it is involved in the response of the skin to UV radiation.

Studies reported that people with certain MC1R variants are at an increased risk of developing melanoma, and that this increased risk is more pronounced in people with CDKN2A mutations. Some of the specific MC1R variants linked to melanoma risk include R151C, R160W, D294H, and D84E [53].

BAP1 is a protein that is encoded by the BAP1 gene. This protein plays a role in the regulation of cell growth and division. It is also involved in the repair of DNA damage. Some studies have suggested that BAP1 may play a role in the development and progression of melanoma. The precise role of BAP1 in melanoma is not yet fully understood. It was suggested that BAP1 may interact with other proteins involved in DNA damage repair, such as TP53 and BRCA1, in the context of melanoma. BAP1 may also interact with signaling pathways that are involved in the regulation of cell growth and division, such as the MAPK and PI3K pathways. 

BAP1-mutated melanomas had distinct clinical and pathological features, including younger age at diagnosis, higher frequency of multiple primary melanomas, uveal melanoma, and a distinct gene expression profile [54].

MITF (microphthalmia-associated transcription factor) is a protein that is encoded by the MITF gene. This gene plays a key role in the development and function of melanocytes, the cells that produce the pigment melanin in the skin. MITF has been identified as an oncogene, meaning that it can promote the development of cancer when it is dysregulated (see Table 3).

In melanoma, MITF may interact with other genes and proteins involved in the development and progression of this cancer. For example, MITF has been shown to interact with the MAPK pathway, which is involved in the regulation of cell growth and division. MITF may also interact with other proteins involved in the regulation of pigmentation, such as MC1R, and with DNA damage repair proteins such as TP53 [55]. 

There are several examples of MITF (microphthalmia-associated transcription factor) variants that have been studied in relation to the risk of melanoma development, a possible new useful marker for the selection of appropriate treatment or as a target for therapy.

One study reported that low MITF expression levels were associated with resistance to chemotherapy in melanoma cell lines. The researchers suggested that MITF downregulation might contribute to the resistance of melanoma cells to cytotoxic drugs [46].

The path of MITF research continues with MITF amplification in melanoma cell lines, which is correlated with increased sensitivity to chemotherapy. The researchers of this study even proposed that MITF amplification might be a predictive marker for chemotherapy sensitivity in melanoma (Table 3).

**Table 3:** Genetic/Somatic alterations present in cutaneous melanoma.

Study	Somatic and Germline Mutation	Gene/Protein Mutations	Cohort/Patients
Casula M. et al. [56]	Germline loss-of-function mutations p16-Leiden mutation 1 mutation in exon 2 of CDK4 1 mutation in exon 2 of CDKN2A Somatic activating mutations MAPK signaling BAP1 pathogenic variants located in the 3p21 region high incidence rate of BRAF somatic mutations (61%)—112 MPM patients pathogenic KIT variant in 4 MPMs Somatic loss-of-function mutations 19 cases TP53 low prevalence of RAS mutations (7%)—28 sporadic MPMs	CDK4 CDKN2A BAP1 BRAF KIT TP53 RAS	2109 CMM (cutaneous malignant melanoma) patients have been followed-up between January 2009 and June 2017 105 (5%) patients had a MPM (Multiple primary melanomas) 102 of them were enrolled in the study
Tan, J.M. et al. [57]	Somatic activating mutations BRAF and NRAS mutation prevalence in acquired naevi by 100% MAPK pathway activation.	BRAF RAS	27 participants *BRAF* and *NRAS* were assessed in 40 globular, reticular, and peripheral rim of globules
Lattanzi, M et al. [58]	Somatic mutations BRAF and NRAS melanoma driver mutations analysed for NM (nodular melanoma) and SSM (superficial spreading melanoma) Eight genes were found to be statistically mutated: NOTCH4, RPS6KA6, BCL2L12, ERBB3, TERT, SNX31, SSPO, and ZNF560 (all *p* < 0.05) three Nmspecific SNVs in ANK3, NOTCH4 and ZNF560 checkpoint blockade immunotherapy (105 NM, 49 SSM) BRAF-targeted therapy (35 NM, 21 SSM)	BRAF RAS NOTCH4, RPS6KA6, BCL2L12, ERBB3, TERT, SNX31, SSPO, ZNF560, ANK3 NOTCH4 and ZNF560	large set of 808 NM and SSM tumors from the most recent SEER database for stage I–III melanoma patients diagnosed from 1973 to 2012 examined a 140-gene panel in a subset of NM and SSM cases using NGS
Sanna, A. et al. [59]	Somatic mutations Oncogenic mutations in CSDhigh and CSDlow frequent mutations in BRAF (n = 35, 49%); V600E and V600K complex hotspot mutations (T599dup and V600_K601delinsE) Mutations in NF1 (n = 12, 17%) and TP53 (n = 17, 24%) 3 cases with hotspot RAC1 mutations	BRAF NF1	cohort of 72 patients (median age from 58–77)
Lebbé, C. et al. [60]	Somatic mutations patients with NRAS-mutated cutaneous melanoma PD-1 blockade either alone or combined with anti-CLTA-4 as first-line therapy	NRAS	191 patients received treatment with Pimasertib, a selective small-molecule MEK1/2 inhibitor Patients from 88 centers in Australia, Europe, Israel, New Zealand, and the USA, from December 2012 to July 2014.
Vergara, I.A. et al. [61]	Somatic mutations 1 BRAF mutant melanoma (ETH-E) 2 NRAS mutant melanomas (SK-G & ETH-J) 3 BRAF/NRAS/NF1 wild-type (MI-F, SK-H, ETH-F Gain and loss of somatic SNVs BRAF identified (n = 8 patients), FAT4 (n = 6), TP53 (n = 4), KMT2C (n = 4), GRIN2A (n = 6), NRAS (n = 2), WRN (n = 4), and ARID2 (n = 4) amongst the most affected genes	BRAF NF1 FAT4, TP53, KMT2C GRIN2A NRAS WRN and ARID2	progressive melanoma from early to late disease in 19 patients two patients (CAS-G and SK-H), metastatic disease was dominated by signature 11 or temozolomide signature, deleterious mutations in DNA-mismatch repair genes, including MSH6 (CAS-G and SK-H), MLH1 (CAS-G), MLH3 (CAS-G and SK-H), and MSH3 (SK-H)
Orlova, K.V. et al. [62]	Somatic mutations BRAF V600E/K was detected in 284 patients (89.9%) rare mutation subtypes V600K, V600D, V600R in 32 patients treatment 1 received combined TT treatment 2 vemurafenib (V) plus cobimetinib (C)	BRAF V600E/K V600K, V600D, V600R	382 patients with advanced BRAF V600 mutant melanoma The patients received targeted therapy (TT) and immunotherapy (IT)
Ascierto, P.A. et al. [63]	Somatic mutations in BRAF combination of cobimetinib plus vemurafenib beneficial long-term effect with BRAF and MEK inhibitor therapy.	BRAF	48 patients in the placebo vemurafenib and 59 patients in cobimetinib
del Carmen Álamo et al. [64]	vemurafenib/cobimetinib between May 2018 and March 2019.	BRAF V600E	41 patients with advanced melanoma
Bobos, M. et al. [65]	Germline mutation inactivation of the tumor suppressor BAP1 in combination with activating BRAF or NRAS mutation Somatic mutations probability of a *BRAF* mutation is significantly higher in patients younger than 40 germline pathogenic mutations in BAP1 gene result in a cancer predisposition syndrome high-CSD melanoma/lentigo maligna melanoma with BRAFV600K being more frequent than BRAFV600E mutations, mutations of *NF1* in 30% and *KIT* increased mutational frequencies in *TP53*, and *ARID2* and cromosomal aberrations immune checkpoint blockade (ICB)	BAP1 BRAF or NRAS BRAFV600K BRAFV600E NF1 and KIT	25 fatal cases of metastatic Spitz melanoma in patients 17 years of age or younger, between 1949 and 2006
Halse H. et al. [66]	Somatic mutations T cell panel (CD8+ T cells, CD4+ T cells and Treg by Flow Cytometry assesment the presence of CD8+PD- 1+IL-7Rα−, CD4+PD-1+, and CD4+OX-40+ T cells indicates activated T cells in the tumor. melanoma cells were the predominant cells present in MelTIL024 and MelTIL026 checkpoint blockade inhibitor anti-CTLA4 (ipilimumab) anti-PD-1 alone (pembolizumab or nivolumab)	CTLA4	Tumor tissue from 21 patients who underwent surgery for stage III (38%) or stage IV (62%) disease.
Heppt, M.V. et al. [67]	Mutations in BRAF and NRAS were identified in 40.1 and 24.4% of cases BRAF was mutated in 87 cases (40.1%), while 53 patients (24.4%) showed NRAS mutations The highest portion of the aggressive NM subtype was observed in NRAS-mutant patients (52.0%) kinase inhibitors immune checkpoint blockade (ICB) with ipilimumab, nivolumab, or pembrolizumab	BRAF or NRAS	melanoma samples of 217 patients with pyrosequencing and Sanger sequencing
Garraway L.A. et al. [55] Müller J. et al. [46]	MITF contribute to melanoma chemoresistance	BRAF NRAS	200 tissue specimens from metastatic melanoma
Wolf Horrell E.M. [68]	MC1R (melanocortin 1 receptor) regulates skin pigmentation, melanoma risk and UV responses	ineffective MSH-MC1R	target drug MSH-MC1R axis for preventing or treating melanoma
Spagnolo F. et al. [51]	CDK4 gene cyclin-dependent kinase 4 inhibits the tumor suppressor RB1	CDK4 gene	promotes proliferation by activation of ERK signaling
Hélias-Rodzewicz Z. et al. [69]	Mutant NRAS melanomas from March 2013 until May 2015 on 267 cutaneous skin melanomas	NRAS Q61	NRAS Q61 mutations in 48 patients (18%) predictor of poorer outcomes
Mitchell S. Stark et al. [70]	MAP3K5 and MAP3K9 mutated in 25% of 8 melanoma cell lines	MAP3K5 and MAP3K9	These mutated genes may contribute to therapy resistance and progression of the disease
Nissan M.H. et al. [71]	NF1, a RAS GTPase activating protein	NF1 (neurofibromin 1) mutations	Loss of NF1 is associated with RAS activation

In some cases, MITF mutations have been observed in melanomas that also harbor BRAF mutations. Some research works suggest that MITF mutations can modulate the response to BRAF-targeted therapies, such as BRAF inhibitors and MEK inhibitors.

On study demonstrated that melanoma cells with MITF amplification were more resistant to BRAF inhibition, while cells with MITF loss were more sensitive. Also, it suggests that MITF may serve as a biomarker to predict the response to BRAF inhibitors [46].

Another study that provides insights into the potential impact of MITF mutations on treatment response in the context of BRAF and NRAS mutations found that melanomas with concurrent NRAS and MITF mutations had distinct gene expression patterns and exhibited resistance to MAPK pathway inhibition. The authors suggested that the presence of MITF mutations might contribute to the resistance of NRAS-mutant melanomas to targeted therapies [46].

### 4.2. Somatic Mutations

In addition to the germline alterations described above, plenty of somatic alterations can occur in numerous genetic loci. Somatic mutations refer to changes or mutations that occur in the DNA of a specific cell or group of cells in the body. These changes can be inherited or acquired during an individual’s lifetime, and they can affect the way that the cells’ function.

In melanoma, somatic alterations can occur in the genes that regulate the growth and division of cells, such as oncogenes and tumor suppressor genes. These changes can lead to uncontrolled growth and division of cells, resulting in the formation of a tumor. Some of the most commonly occurring somatic alterations in melanoma include mutations in the BRAF, NRAS, and KIT genes. Other genetic changes that have been identified in melanoma include alterations in the TP53, PTEN, and CDKN2A genes.

These genetic changes can be used to classify different subtypes of melanoma and to develop targeted treatments that are specific to the genetic changes present in an individual’s tumor [72].

The RAS-RAF-MEK-ERK-MAPK pathway is a signaling pathway that is involved in cell growth and division. It is activated by a variety of stimuli, including growth factors and other signaling molecules, and it plays a critical role in the development and progression of many types of cancer, including melanoma. 

Targeted therapies that inhibit this pathway, such as BRAF inhibitors, have been developed to treat melanoma and other cancers that have activating mutations in this pathway.

BRAF V600E, the most common BRAF mutation in melanoma (40–50% of cases), occurs when a single amino acid (valine) is replaced with another amino acid (glutamic acid) at position 600 in the BRAF protein. This mutation leads to the constant activation of the RAS-RAF-MEK-ERK-MAPK pathway and is associated with a more aggressive form of melanoma.

The first discovery of the BRAF V600E mutation in 2022 by Dr. Garraway and Dr. Chin, and later on, its significance in melanoma treatment has been a major breakthrough in this field of oncology.

Clinical phase III trials like the BRIM-3 trial, BREAK-3, COMBI-d, COMBI-v, and COLUMBUS are significant examples on the full list of trials focused on the treatment of melanoma with the BRAF V600E mutation.

Resistance to BRAF inhibitors is a challenge in the treatment of metastatic melanoma. Researchers are investigating strategies to overcome resistance mechanisms, including the development of new targeted therapies and combination approaches. Preclinical and clinical studies are exploring the use of different agents and combinations to delay or overcome resistance, such as adding novel MAPK pathway inhibitors or combining BRAF inhibitors with other targeted therapies.

One of these studies identified the mechanisms of acquired resistance to the BRAF inhibitor PLX4032 (vemurafenib). The researchers found that secondary mutations in the BRAF gene, specifically the BRAF V600E/K to NRAS Q61R mutation, were associated with resistance to BRAF inhibitors.

Another example of acquired resistance is by activation of the MAPK pathway through genetic alterations, including mutations in NRAS, MEK1, and MEK2, as well as amplification of BRAF or MEK1/2 genes, as common mechanisms of resistance.

Overcoming resistance to BRAF inhibitors in melanoma is a complex challenge, but researchers have been investigating various strategies to address this issue. 

One of the first tested and effective strategies is combining BRAF inhibitors with MEK inhibitors, leading to improved response rates and delayed resistance compared to BRAF inhibitor monotherapy like in COMBI-d and COMBI-v trials, but resistance to BRAF inhibitors often involves the activation of parallel signaling pathways [73,74,75,76,77].

Targeting parallel pathways can be an effective approach. For example, combining BRAF inhibitors with inhibitors targeting the PI3K/AKT/mTOR pathway or the receptor tyrosine kinase pathway (such as EGFR or MEK inhibitors) has shown promise in preclinical studies.

In some cases, resistance to BRAF inhibitors is driven by alterations in upstream or downstream components of the MAPK pathway. Targeting these alterations with specific inhibitors or combination therapies can overcome resistance.

Combining BRAF inhibitors with immune checkpoint inhibitors has shown synergistic effects and improved outcomes, particularly in patients with resistance to BRAF inhibitors.

Additionally, strategies such as combining targeted therapies with immunotherapies, epigenetic modulators, or nanoparticle-based delivery systems are under investigation.

Biomarkers can help identify specific mechanisms of resistance and guide treatment decisions. For example, the presence of specific mutations or alterations (e.g., NRAS mutations) can inform the selection of targeted therapies or combination strategies [75,76,77].

BRAF V600K mutation occurs when valine at position 600 in the BRAF protein is replaced with lysine. It is less common than the V600E mutation, occurring in approximately 5–10% of melanomas. While both V600E and V600K mutations result in abnormal activation of the BRAF protein, studies have shown that the V600K mutation may confer different biological properties and potentially reduce the sensitivity of BRAF inhibitors. As a result, treatment responses may be less favorable in patients with the V600K mutation compared to those with the V600E mutation [73,74].

Mutations in the NRAS gene are reported, as NRAS is a member of the RAS family of proteins and is activated by growth factors and other signaling molecules. 

Activating mutations in the NRAS gene have been identified in melanoma and can lead to the constant activation of the RAS-RAF-MEK-ERK-MAPK pathway.

There are several different types of somatic mutations that can occur in the NRAS gene, including point mutations, insertion and deletion mutations, and amplification of the gene.

Some of the most frequently occurring NRAS mutations in melanoma include Q61K, Q61R, and Q61L. These mutations result in activation of the NRAS protein and can lead to uncontrolled cell growth and proliferation, contributing to the development and progression of melanoma. Other NRAS mutations, such as G12S and G13D, have also been identified in melanoma, although they are less common [78,79,80].

One study evaluated the use of the MEK inhibitor binimetinib in patients with NRAS-mutated melanoma. The study reported favorable response rates in this patient population [52].

KRAS is a member of the RAS family of proteins and is activated by growth factors and other signaling molecules. Activating mutations in the KRAS gene have been identified in melanoma and can lead to the constant activation of the RAS-RAF-MEK-ERK-MAPK pathway. Some of the most frequently occurring KRAS mutations in melanoma include G12C, G12V, and G12D. These mutations result in activation of the KRAS protein and can lead to uncontrolled cell growth and proliferation, contributing to the development and progression of melanoma. Other KRAS mutations, such as Q61K and Q61R have also been identified in melanoma, although they are less common [81].

MAPK (mitogen-activated protein kinase) pathways are signaling pathways that transmit signals from extracellular stimuli to the interior of cells. MAPK pathways are activated by a variety of stimuli, including growth factors, hormones, and stress signals, and they play important roles in many cellular processes, including cell proliferation, differentiation, and survival.

There are several different types of MAPK pathways, including the ERK (extracellular signal-regulated kinase) pathway, the JNK (c-Jun N-terminal kinase) pathway, and the p38 pathway. Each of these pathways consists of a series of proteins that transmit the signal from the cell surface to the interior of the cell, ultimately leading to changes in gene expression and other cellular responses.

Two mutations in MAPK pathways can play a role in the development of melanoma, a type of skin cancer. One of these mutations is a mutation in the BRAF gene, which encodes a protein that is a key component of the MAPK pathway. This mutation leads to the activation of the MAPK pathway, which can promote the proliferation and survival of cancer cells. The other mutation is in the NRAS gene, which encodes another protein that is involved in the MAPK pathway. This mutation also activates the MAPK pathway and can contribute to the development of melanoma.

There are several targeted therapies that have been developed to specifically target MAPK pathway mutations in melanoma, including BRAF inhibitors and MEK inhibitors. These therapies can be effective at slowing the growth of melanoma cells and may be used as part of the treatment for patients with this type of cancer.

MAP2K1, MAP2K2, MAP3K5, and MAP3K9 are proteins that are involved in the MAPK pathway. They act as enzymes that transmit signals from extracellular stimuli to the interior of cells, ultimately leading to changes in gene expression and other cellular responses. Mutations in MAP2K1 and MAP2K2 can affect the function of these proteins, and may contribute to the development of cancer [81,82].

There have been several studies that have investigated the role of somatic mutations in MAP2K1 and MAP2K2, and in MAP3K5 and MAP3K9 in melanoma. These studies have found that mutations in these genes can occur in melanoma and may contribute to therapy resistance and progression of the disease [52]. Some studies have also shown that targeting MAP2K1 and MAP2K2, and MAP3K5 and MAP3K9 with specific drugs can be effective at inhibiting the growth of melanoma cells in preclinical models [70,82,83].

In addition, the siRNA-induced reduction of MAP3K9 expression in cells treated with the chemotherapeutic agent temozolomide translated into a rise in viability when compared to cells treated with temozolomide alone, indicating that reactivation of MAP3K9/MAPK3K5 signaling pathways may have therapeutic implications [70,82,83].

KIT is a receptor tyrosine kinase that is activated by the binding of a growth factor called stem cell factor. Activating mutations in the KIT gene have been identified in melanoma and can lead to the constant activation of the RAS-RAF-MEK-ERK-MAPK pathway. C-KIT is a receptor tyrosine kinase that acts as a receptor for stem cell factor (SCF) ligand, and c-KIT-SCF signaling is required for melanocytic development. Mutations in the c-KIT gene are uncommon in melanoma, with an incidence of approximately 1–2%. However, when they do occur, the most frequent specific somatic mutations in the c-KIT gene in melanoma include KIT D816V mutation.

D816V is important as this mutation involves the substitution of a valine amino acid for an aspartic acid at position 816 of the c-KIT protein. However, in melanoma, the D816V mutation in the KIT gene is much less common and is typically associated with acral or mucosal melanomas. Unfortunately, KIT inhibitors like imatinib have limited efficacy in melanoma cases with the D816V mutation. This mutation tends to confer resistance to KIT inhibitors, making these treatments less effective. While imatinib and, most recent nilotinib may have some activity against other KIT mutations in melanoma, such as L576P or V559D, the D816V mutation is generally regarded as less responsive to these targeted therapies. In cases of advanced melanoma with KIT mutations, alternative treatment strategies, such as immune checkpoint inhibitors or other targeted therapies may be considered [84,85].

Somatic NF1 (neurofibromin 1) mutations are observed in a subset of melanoma cases, with varying frequencies reported across studies. The prevalence of NF1 mutations in melanoma ranges from approximately 10% to 25%, depending on the specific cohort and the detection methods used.

Many somatic mutations, like nonsense mutations, frameshift mutations, splice site mutations or large deletions in the gene were identified in melanoma, resulting in loss-of-function of the NF1 protein.

This leads to the dysregulation of downstream signaling pathways, such as the Ras-MAPK pathway promoting cell growth and survival [86].

Somatic NF1 mutations often co-occur with other genetic alterations, particularly BRAF and NRAS mutations, which are common in melanoma. The concurrent presence of NF1 mutations with BRAF or NRAS mutations may influence disease progression and response to targeted therapies [87].

Emerging evidence suggests that somatic NF1 mutations in melanoma may impact the tumor microenvironment and immune response. NF1 loss-of-function mutations have been associated with increased tumor immunogenicity, infiltration of immune cells, and potential responsiveness to immunotherapies [71].

ERBB4 gene has been found to be mutated in a variety of cancer types, including melanoma. The most common specific somatic mutations in ERBB4 in melanoma are usually point mutations, which are changes to a single nucleotide (a building block of DNA) in the gene’s sequence. Some examples of specific somatic mutations in ERBB4 that have been identified in melanoma include the p. Glu1387Lys mutation, which involve a change of the amino acid glutamate to lysine at position 1387 in the protein. It has been found to be present in about 1% of melanoma tumors.

Recent findings show that the prevalence of ERBB4 mutations in melanoma is relatively low compared to other genetic alterations like BRAF or NRAS mutations.

As a result, there is a scarcity of dedicated clinical studies investigating targeted therapies for ERBB4-mutated melanoma [88,89,90].

PTEN (Phosphatase and Tensin Homolog) is a gene that encodes the protein PTEN. PTEN is a well-known tumor suppressor protein that plays a crucial role in regulating cell growth, division, and survival.

Genetic alterations in the PTEN gene can occur in various ways, including point mutations, deletions, insertions, and rearrangements. These alterations can lead to the loss or reduction of PTEN protein function, resulting in PTEN deficiency or inactivation.

Mutations or deletions in PTEN are associated with several types of cancers, including melanoma.

PTEN acts as a tumor suppressor by negatively regulating the PI3K/AKT signaling pathway, which is involved in cell growth, survival, and metabolism. Loss of PTEN function leads to increased PI3K/AKT signaling, promoting cell proliferation and survival, and potentially contributing to melanoma growth and progression.

Also, PTEN alterations in melanoma may be associated with specific clinical and pathological features. For example, PTEN loss has been correlated with thicker primary melanomas, advanced disease stage, and poorer prognosis.

Preclinical studies have shown that PTEN deficiency can confer resistance to certain targeted therapies and immunotherapies, highlighting the importance of considering PTEN status when selecting treatment options for melanoma patients. They also found that combining BRAF inhibitors with immune checkpoint inhibitors restored T cell infiltration and improved treatment response in PTEN-deficient melanoma models [91,92].

The TERT gene, also known as Telomerase Reverse Transcriptase, encodes the catalytic subunit of telomerase, an enzyme involved in maintaining the length of telomeres.

Genetic alterations in the TERT gene have been extensively studied in melanoma.

One of the most common genetic alterations in the TERT gene in melanoma is the presence of mutations in the TERT promoter region. The C228T and C250T TERT promoter mutations are frequently found in melanoma cases. These mutations result in increased TERT expression and activation of telomerase, leading to the maintenance of telomere length and enhanced cell proliferation. Multiple studies have reported a high prevalence of TERT promoter mutations in melanoma, particularly in cases with aggressive clinical features and poor prognosis.

TERT promoter mutations are associated with clinicopathological characteristics of melanoma like advanced tumor stage, increased tumor thickness, ulceration, and metastasis. These mutations have even a prognostic value, since TERT promoter mutations have been identified as independent prognostic markers in melanoma. It is associated with worse survival outcomes, indicating their potential as prognostic biomarkers in clinical practice. Studies have also explored the relationship between TERT promoter mutations and other common genetic alterations in melanoma, such as BRAF and NRAS mutations. Some investigations have reported mutually exclusive patterns, suggesting that TERT promoter mutations may represent an alternative pathway of telomerase activation in melanoma cases lacking BRAF or NRAS mutations. Also, it has been described as even having potential therapeutic implications. Targeting telomerase and the TERT gene has been explored as a potential therapeutic strategy in melanoma. Preclinical studies have shown that inhibiting telomerase activity or TERT expression can lead to telomere shortening, cellular senescence, and reduced melanoma cell proliferation. However, clinical translation of these findings is still under investigation [93,94].

Technological advancements in genetic testing and sequencing have significantly improved our ability to identify somatic and germline changes associated with melanoma. These advancements have expanded our understanding of the hereditary risk for melanoma, facilitated genetic risk stratification, and influenced personalized management and treatment approaches. Continued advancements in genomic technologies will undoubtedly contribute to further unraveling the complex genetic landscape of melanoma and improving patient outcomes.

### 4.3. Non Coding Regions

Recent efforts have focused on exploring non-coding regions of the genome, including regulatory elements and non-coding RNAs, to better understand their contribution to melanoma development and hereditary risk. Techniques like chromatin immunoprecipitation sequencing (ChIP-seq) and RNA sequencing (RNA-seq) have shed light on the role of non-coding genomic alterations in melanoma progression and treatment response.

In the context of melanoma, miRNAs have gained significant attention as potential biomarkers and therapeutic targets due to their involvement in melanoma initiation, progression, and metastasis.

The dysregulation of miRNAs in melanoma can occur through various mechanisms, including genetic alterations, epigenetic modifications, and changes in their processing machinery. Altered miRNA expression profiles have been observed in melanoma compared to normal skin, and specific miRNAs have been identified as oncogenes or tumor suppressors based on their effects on melanoma cell growth, invasion, and immune response.

MiRNAs in melanoma are involved in the regulation of several key signaling pathways, including the MAPK pathway (e.g., miR-let-7a, miR-137, miR-184, miR-211), PI3K/AKT pathway (e.g., miR-17-5p, miR-126, miR-155), and Wnt/β-catenin pathway (e.g., miR-26a, miR-26b, miR-146a). They can modulate the expression of target genes involved in these pathways, affecting cellular processes critical for melanoma development and progression.

Furthermore, miRNAs have been implicated in melanoma immune evasion mechanisms. Some miRNAs can regulate the expression of immune checkpoint molecules such as PD-L1, CTLA-4, and TIM-3, thereby influencing the immune response against melanoma cells. Dysregulated miRNAs in melanoma can also impact the tumor microenvironment, including the regulation of angiogenesis, inflammation, and extracellular matrix remodeling [56,57,58].

Due to their stability and detectability in various biological samples, miRNAs hold great promise as non-invasive biomarkers for melanoma diagnosis, prognosis, and prediction of treatment response. The identification of specific miRNA signatures associated with different melanoma subtypes, disease progression, and therapy resistance has the potential to guide personalized treatment decisions and improve patient outcomes.

Moreover, the therapeutic potential of miRNAs in melanoma is being explored. Strategies to restore or inhibit specific miRNAs using synthetic miRNA mimics or anti-miRNA oligonucleotides have shown promise in preclinical studies, offering a novel approach to modulate gene expression and target melanoma cells specifically.

## 5. Conclusions

Melanoma is an extremely dangerous illness. It is a diverse and complicated condition that can be difficult to detect and treat. Understanding the processes that cause melanomagenesis and allow melanomas to avoid the immune system will help us to develop better diagnostic and treatment options for this illness.

Considering the complicated reprogramming of cell death and survival pathways during melanomagenesis, a single “magic bullet” treatment for melanoma seems improbable. Effective treatment(s) will very certainly entail a mix of numerous medications directed at distinct resistance mechanisms. Despite their numerous genetic and epigenetic changes, melanoma cells continue to express proteins involved in the last stages of apoptosis. As previously stated, techniques aimed especially at exploiting this characteristic and killing melanoma cells by bypassing or overcoming upstream death defects have yielded promising results in experimental models. These studies are being transferred into clinical settings, and we should be enthusiastic about science’s long-awaited victory against this virulent illness.

## Figures and Tables

**Table 1:** Targeted Therapy, individual/combined in melanoma treatment.

No	Targeted Therapy	Clinical Trials	Study
1	Dacarbazine 1970	- In combination with BRAF inhibitors	Lui P., [11], 2007
2	Interferon alpha-2b 1995	- Phase III ECOG 1684 trial	Dummer R, [12], 2006
3	Vemurafenib, a BRAF inhibitor 2011	- BRIM-3 study	Kim A, [13], 2016
4	Dabrafenib, another BRAF inhibitor, and trametinib, a MEK inhibitor 2013	- BREAK-3 and METRIC trials	Planchard D, [14], 2016
5	Ipilimumab, the first immune checkpoint inhibitor 2013	- MDX010-20 study	Weber JS, [15], 2013
6	Pembrolizumab and nivolumab 2014	- KEYNOTE-001 and CheckMate-066 studies	Nanda VGY, [16], 2016
7	Oncolytic viruses T-VEC, talimogene laherparepvec	- OPTiM (Oncovex Pivotal Trial in Melanoma)	Ribas A, [17], 2017 Andtbacka RHI, [10], 2019
8	Immune checkpoint inhibitors, such as anti-PD-1 antibodies (e.g., pembrolizumab, nivolumab) and anti-CTLA-4 antibodies (e.g., ipilimumab)	- Combination therapy - CheckMate-067 studies	Carlino MS, [18], 2021
9	Anti-LAG-3 antibody with nivolumab	- Combination therapy for solid tumors	Koseła-Paterczyk H, [19], 2023
10	Anti-PD-1/PD-L1 antibodies with anti-LAG-3 (lymphocyte-activation gene 3) antibodies	- New combination therapy - NCT01968109	Tawbi HA, [20], 2022 Huuhtanen J, [21], 2023
11	Combination of miR-34a with ipilimumab Combination of miR-34a with vemurafenib	- Simultaneous inhibition of miRNAs using specific inhibitors reduced the CCL2 release - No effect when inhibiting each individual miRNA	Vergani E, [22], 2016
12	CAR-T cells 24 metastatic melanoma patients	- NCT01218867, 2010–2019	Yu, J. [23], 2018 Soltantoyeh T, [24], 2021
13	MAGE-A3 immunotherapeutic	- DERMA was a phase 3, double-blind, randomised, placebo-controlled trial on 1345 patients - no clinical benefit was recorded	Dreno B, [25], 2018

**Table 2:** Pathology of melanoma.

No	Melanocytic Marker	Detection, Prognostic or Proliferation of Melanoma	Study
1	S-100 protein with different isoforms: S-100A1, S-100A2, and S-100A4 Subtypes: S100B, S100P, S100A4, S100A6, and S100A13	- detect a possible melanocytic neoplasm	[38,18]
2	HMB-45 ‘human melanoma black,’ identified in 1986	- detects a melanosomal glycoprotein (Pmel17)	[39,40]
3	MART-1—Melan A	- histopathological marker for melanocytic progenitors	[40,41]
4	Ki67 cell cycle regulatory protein	- a greater Ki67 level, worse prognosis	[42,43]
5	Tyrosinase	- strongly positive IHC in 84% of melanoma cases - TYR negatively regulates vasculogenic mimicry which leads to antioncogenic function of TYR in melanomas (Kamo H, 2022).	[44,45]
6	MITF (microphthalmia-associated transcription factor)	- a low MITF/AXL ratio = early resistance to multiple targeted drugs [46]	[46,47]
